# Chromosomal Localization and Contribution of Three Homoeologous Genes to Biosynthesis of Cytosolic Aspartate Aminotransferase in Common Wheat

**DOI:** 10.1007/s40011-015-0536-7

**Published:** 2015-05-27

**Authors:** Marcin Maciąga, Michał Szkop, Andrzej Paszkowski

**Affiliations:** Department of Biochemistry, Faculty of Agriculture and Biology, Warsaw University of Life Sciences – SGGW, Nowoursynowska 159, 02-776 Warsaw, Poland

**Keywords:** Allozymes (Isozymes), Aneuploid and deletion lines of wheat, Aspartate aminotransferase, Chromosomal localization, Common wheat (*Triticum aestivum*)

## Abstract

Chromosomal localization of the three homoeologous genes encoding cytosolic aspartate aminotransferase in common wheat (*Triticum aestivum* cv. Chinese Spring, 2n = 6x = 42, AABBDD) was specified to: 3AL (0.42÷0.61), 3BL (0.38÷0.41) and 3DL (0.23÷0.81) by a comparative zymographic analysis of the enzymatic activities in deletion lines. It was also attempted to precisely explain the nature of the relationship between a number of genes encoding α and β subunits and a distribution of staining intensity of cytosolic aspartate aminotransferase allozyme activity bands using aneuploid lines of common wheat with modified third pair of homoeologous chromosomes from genomes A, B and D, on which the genes encoding subunit α (genome A) and β (genome B and D) are localized. The highest consistency between the experimental results and the theoretical distributions was achieved by substituting values of α = 0.57 and β = 0.43 in a theoretical model. These results demonstrate that the individual participation of the diploid genome A in the biosynthesis of the cytosolic aspartate aminotransferase allozymes subunits is greater than the individual participation of the diploid genomes B and D.

## Introduction

Aspartate aminotransferase (AAT; EC 2.6.1.1) catalyses the fully reversible transamination reaction that occurs between l-aspartate and 2-oxoglutarate with the formation of oxaloacetate and l-glutamate. In plants, AAT isozymes are located in different subcellular compartments such as cytosol, mitochondria, plastids or glyoxysomes and play a pivotal role in regulation of carbon and nitrogen flux [1]. It is therefore not surprising that first attempts have been made to obtain transgenic plants with increased level of AAT activity [2–4].

There are usually three enzymatic zones (isozymes) observed on the zymograms of AAT from common wheat and these zones are composed of cytosolic, mitochondrial and plastidial allozymes [5–7]. Similar pattern was observed in case of different plants closely related to wheat as: maize [8], rice [9], millet [10], barley [11, 12] or finger millet [13]. Based on the zymographic analyses of AAT preparations from different subcellular fractions of wheat seedlings, it was found that AAT-1 zone (the one with the highest electrophoretic mobility towards the anode) is composed of mitochondrial allozymes, while AAT-2 and AAT-3 zones correspond to plastidial and cytosolic allozymes, respectively [7]. Hart [14] using aneuploid lines of common wheat found that genes encoding allozymes from AAT-3 zone are localized on the long arms of the third pair of homeologous chromosomes from the diploid A, B and D genomes—3AL, 3BL i 3DL. Derivation of wheat deletion lines [15] allowed more precise localization of the genes encoding cytosolic allozymes on the chromosome. Using such deletion lines Qi et al. [16] established the localization of 7,104 expressed sequence tags (EST), including BF473016 EST which is a fragment of the sequence exhibiting a high degree of identity to the sequence of the genes encoding cytosolic AATs in different plants. It was mapped on the 3AL (0.42÷0.78), and on the short arms of chromosomes in genome B and D, i.e. 3BS (0.57÷1.00) and 3DS (0.24÷1.00) (ibid.). The numbers in brackets represent a part of a chromosome arm where a specific fragment of the mapped nucleotide sequence is localized. It should be noted that there is some contradictory data regarding chromosomal localization of the AAT genes that needs to be clarified. According to Hart [14] genes encoding cytosolic AAT allozymes are located on 3AL, 3BL and 3DL, but by EST mapping method these genes were localized on 3AL (0.42÷0.78), 3BS (0.57÷1.00) and 3DS (0.24÷1.00) [16].

Hart et al. [17] using zymography analyzed the composition of AAT-3 zone from common wheat and demonstrated that this zone is composed of three allozymes. Assuming that AAT is enzymatically active only in the form of a dimmer, the authors proposed the following subunit composition of the allozymes from AAT-3 zone: band at the AAT-3a position (positioned closest to the anode) is formed from ββ subunits, AAT-3b from αβ subunits and AAT-3c from αα subunits. The bands at the AAT-3a and AAT-3b positions were the most intensive on the zymograms. This indicated according to the authors that there were twice more β subunits encoded by genomes B and D than α subunits encoded by genome A. It should be noted that Hart et al. [17] evaluated the distribution of the staining intensity of AAT allozymes activity bands only qualitatively. Maciąga and Paszkowski [7] using zymography analyzed quantitatively the occurrence frequency of α and β subunits in common wheat and calculated that the frequency of α and β subunits were 0.62 and 0.38, respectively. There was, however, only one line of common wheat used in this study, while there are currently available different aneuploid wheat lines with various combinations within homoeologous chromosomes.

Thus, the aim of the present study was to specify the chromosomal localization of the genes encoding cytosolic AAT allozymes, and to determine precisely the frequency of the α and β subunits composing these allozymes. For this purpose a comparative zymographic analysis of the AAT activities in deletion and aneuploid lines of common wheat was applied. The authors’ intention was to enrich the present knowledge about the participation of a particular diploid genomes in biosynthesis of proteins in polyploid plants. This study was also aimed to experimentally complement, using a conventional biochemical approach, the wheat genome sequencing project exploring the genome of this economically important plant [18].

## Material and Methods

### Plant Material and Isolation of AAT

Monosomics (M3A, M3B), ditelosomics (Dt3AL, Dt3AS, Dt3BS), nullisomic–tetrasomics (NT3B-3A) and deletion lines (del3AL-01, -03, -04, -05, -06, -08; del3BL-01, -02, -03, -04, -06, -07, -08, -09, -10, -11; del3DL-01, -03) of common wheat (*Triticum aestivum* cv. Chinese Spring, 2n = 6x = 42, AABBDD) were used in the present study. Seedlings were grown in universal soil (pH 6.5) in a growth chamber set on a 16 h light (400 μE, 22 °C) and 8 h dark (18 °C) photoperiod. Green sections of the 14 days old wheat seedlings were blended in homogenizer in 100 mmol L^−1^ Tris–HCl buffer pH 7.5 and centrifuged at 12,000×*g* for 10 min. Obtained supernatants were used for AAT activity assay and for a native PAGE.

### Native PAGE and AAT Activity Assay

AAT activity was assayed according to Bergmayer and Bernt [19]. The reaction mixture consisted of 100 mmol L^−1^ Tris–HCl buffer pH 7.5, 200 mmol L^−1^ l-aspartate, 10 mmol L^−1^ 2-oxoglutarate, 0.12 mmol L^−1^ NADH and 0.4 U mL^−1^ malate dehydrogenase. Approximately 50 mU of enzyme activity was loaded to the electrophoretic wells. Native PAGE was performed according to Laemmli [20] using 7.5 % resolving and 4 % stacking gel. Electrophoresis (30 mA per plate) was run in 50 mmol L^−1^ Tris–glycine buffer pH 9.1 for 180 min. Zymograms were visualized according to Stejskal [21] by incubating gels in reaction mixture containing 100 mmol L^−1^ Tris–HCl buffer pH 7.5, 8 mmol L^−1^ l-cysteine sulfate, 5 mmol L^−1^ 2-oxoglutarate, 0.1 mmol L^−1^ pyridoxal-5′-phosphate, 0.5 mmol L^−1^ 3-(4,5-dimethylthiazol-2-yl)-2,5-diphenyl-tetrazolium bromide and 0.16 mmol L^−1^ *N*-methylphenazonium methosulfate. Scanned zymograms were analysed densitometrically using ImageJ software.

### Statistical Approach

Taking into account that each AAT allozyme is composed of two subunits (αα, αβ or ββ), of which the α subunit is biosynthesized by the diploid genome A and the β subunit is biosynthesized by the diploid B and D genomes, equation (*xα* + *yβ*)^2^ was used to derive appropriate formula for a particular aneuploid line. The amount of loci encoding each subunit is represented by x and y in the equation. Subsequently, by substitution of various frequencies of the α and β subunits (*α* + *β* = 1) in the derived formulae (i.e. 0 αα:0 αβ:16 ββ for Dt3AS line, 1 αα:8 αβ:16 ββ for M3A line, 4 αα:12 αβ:9 ββ for M3B line, 1 αα:4 αβ:4 ββ for Dt3AL line, 1 αα:2 αβ:1 ββ for Dt3BS line and 4 αα:4 αβ:1 ββ for NT3B-3A line) many theoretical distributions of the staining intensity of AAT-3 allozymes activity bands were obtained. The statistical distance between the theoretical and the experimental distributions (both expressed in %) was measured using the χ^2^ function, i.e. the difference between the densitometric measurement and the expected value for a single band was squared and divided by the expected value. Then the obtained values for all of the bands were added up. Lower χ^2^ values indicate better fit between the theoretical and the experimental distributions of staining intensity of AAT-3 allozymes activity bands.

## Results and Discussion

**Fig. 1:**
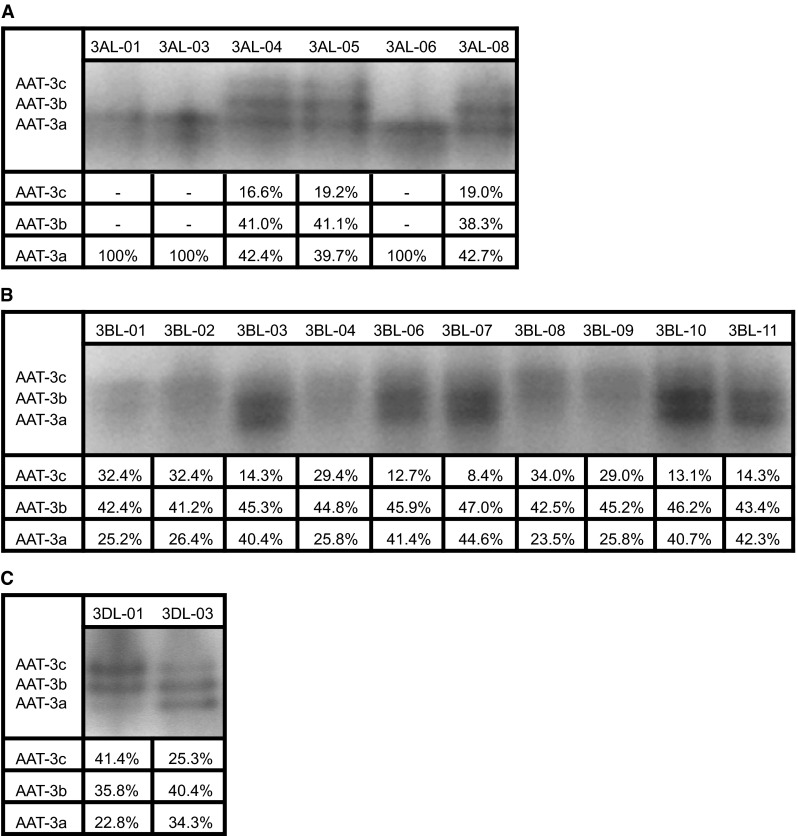
Zymographic analysis of the cytosolic AAT allozyme activities in deletion lines of common wheat (*Triticum aestivum*) with modified third pair of homologous chromosomes within a diploid genome A (**a**), genome B (**b**) and genome D (**c**) together with calculated experimental distributions of the staining intensities of AAT-3a, -3b and -3c activity bands. Calculated distributions are the means of four independent densitometric measurements of the scanned gel. Standard deviations were within ±3 % of the mean

In order to specify chromosomal localization of the genes encoding α subunit of the cytosolic AAT allozymes, the following deletion lines of common wheat were used: del3AL-01, -03, -04, -05, -06, -08. Zymographic analysis of the AAT activities in these lines revealed that there were three bands observed on the zymogram for del3AL-04 (0.61), del3AL-05 (0.78) and del3AL-08 (0.85) lines, while for del3AL-01 (0.26), del3AL-03 (0.42) and del3AL-06 (0.21) lines only a single band occurred on the zymogram (Fig. 1a). It indicates that del3AL-01, del3AL-03 and del3AL-06 lines lack a chromosome fragment on which the genes encoding α subunit are localized, and they encode only β subunits forming a single band at the AAT-3a position. In lines del3AL-04, del3AL-05 and del3AL-08 there are chromosomes without a fragment of chromatin, but still with genes encoding the α subunit. Therefore, there are two types of subunits formed and there are three bands visible on the zymograms of these lines.

Localization of the genes encoding β subunit on the long arms of the third pair of homologous chromosomes of the diploid genome B appeared to be more difficult, because β subunit is encoded also by genome D. Therefore, for all of the studied deletion lines (i.e. del3BL-01, -02, -03, -04, -06, -07, -08, -09, -10, -11) there were three bands of AAT activity visible on the zymogram within cytosolic AAT zone, and these bands differed only in the intensity of staining (Fig. 1b). However, it can be observed that for lines del3BL-01 (0.31), del3BL-02 (0.22), del3BL-04 (0.07), del3BL-08 (0.28) and del3BL-09 (0.38) the bands on the zymogram at the AAT-3c (αα) and AAT-3b (αβ) positions are more intensive than bands at the AAT-3a (ββ) positions. This suggests that these lines lack a fragment of chromatin from the long arm of the chromosome on which the genes encoding β subunit are localized, and therefore the β subunit is encoded only by genome D. In the other deletion lines: del3BL-03 (0.41), del3BL-06 (0.54), del3BL-07 (0.63), del3BL-10 (0.50) and del3BL-11 (0.81) there are two α subunits encoded by the genome A per four β subunits encoded by the genome B and D. As a result the bands on the zymogram at the AAT-3a (ββ) i AAT-3b (αβ) positions are the most intensive.

Only two wheat deletion lines were available, in which the deletions concern the long arms of the third pair of homologous chromosomes in the genome D: del3DL-01 and del3DL-03. Zymographic analysis of the AAT activities in these lines revealed that for del3DL-01 (0.23) line the bands at the AAT-3c (αα) and AAT-3b (αβ) positions were more intensive than the band in the AAT-3a (ββ) position. In turn, for del3DL-03 (0.81) line the most intensive staining was observed for bands at AAT-3a (ββ) and AAT-3b (αβ) positions (Fig. 1c).

**Fig. 2:**
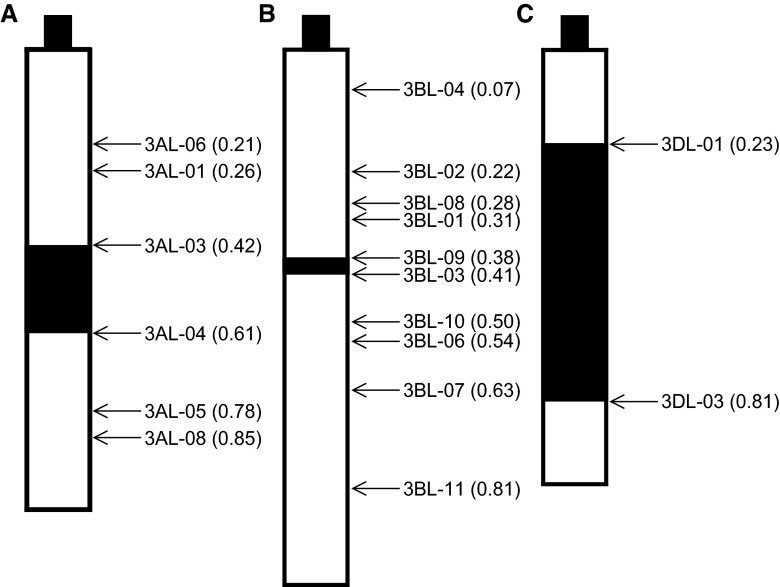
Ideogram of the long arm of the third pair of homologous chromosomes within a diploid genome A (**a**), genome B (**b**) and genome D (**c**) of common wheat (*Triticum aestivum*). Chromosomal localizations of the genes encoding α and β subunits are marked with a *black color*

All these results, taken together, allow to claim that the genes encoding cytosolic AAT allozymes are located on the long arms of the third pair of homologous chromosomes in the region of 0.42–0.61 in the genome A (Fig. 2a), 0.38–0.41 in the genome B (Fig. 2b), and 0.23–0.81 in the genome D (Fig. 2c). It completely confirms and simultaneously specifies the localization of these genes found by Hart [14]. In turn, it rules out the chromosomal localization of the two cytosolic AAT genes on the short arms of the third pair of homeologous chromosomes from the genome B and D reported by Qi et al. [16]. It is worth noting that the results presented in the present study were affirmed using bioinformatics tools. Currently wheat genome is sequenced in over 90 %, and a great part of the data including the sequence of the third pair of homeologous chromosomes became recently available. Searching of Ensembl Plants database (http://plants.ensembl.org/index.html) using the cytosolic AAT gene sequence (Gene ID: EU346759) [22] resulted in the identification of three genes exhibiting respectively 99 % (Gene ID: TRAES3BF023000050CFD_g), 98 % (Gene ID: traes_3AL_B137CC856) and 97 % (Gene ID: traes_3DL_870617108) sequence identity to the query sequence. Identified genes are assigned to the third pair of homologous chromosomes in the genome B, and to the long arms of the third pair of homologous chromosomes in the genomes A and D, respectively. The sequence identity between the transcripts of these genes ranges from 97 to 98 %.

**Fig. 3:**
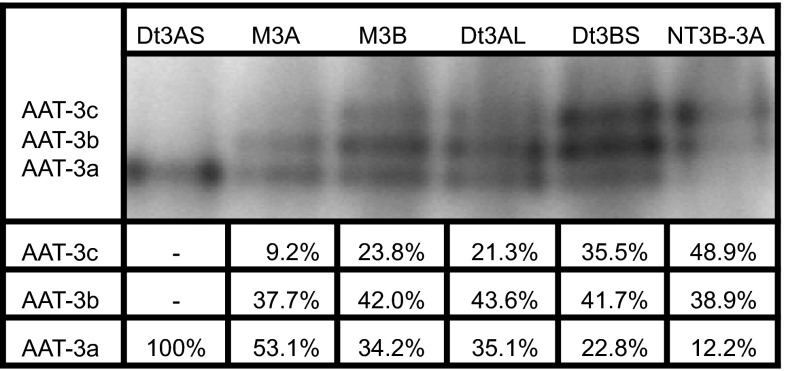
Zymographic analysis of the cytosolic AAT allozyme activities in different aneuploid lines of common wheat (*Triticum aestivum*) together with calculated experimental distributions of the staining intensities of AAT-3a, -3b and -3c activity bands. Calculated distributions are the means of four independent densitometric measurements of the scanned gel. Standard deviations were within ±1 % of the mean

In the second part of the present study it was attempted to precisely explain the nature of the dependence between the number of the genes encoding α and β subunits, and the distribution of the staining intensity of the AAT-3 allozymes activity bands. For this purpose the following aneuploid wheat lines with modifications within the third pair of homologous chromosomes in the diploid genomes A, B and D were used: Dt3AS, M3A, M3B, Dt3AL, Dt3BS, NT3B-3A. Dt3AS (0*α* + 4*β*)^2^ is a ditelosomic line which lacks the long arms of the third pair of homologous chromosomes in diploid genome A, on which the genes encoding α subunits are localized, and therefore this plant synthesizes only β subunits. M3A (1*α* + 4*β*)^2^ is a monosomic line that lacks one chromosome of the third pair of chromosomes in genome A and synthesizes four β subunits per one α subunit. The monosomic M3B (2*α* + 3*β*)^2^ line lacks one chromosome of the third pair in the diploid genome B and synthesizes three β subunits per two α subunits. Dt3AL (2*α* + 4*β*)^2^ is a ditelosomic line which lacks short arms of the third pair of homologous chromosomes in the diploid genome A. The AAT genes are not localized on this arms, so this line synthesizes four β subunits per two α subunits. The ditelosomic Dt3BS (2*α* + 2*β*)^2^ line lacks long arms of the third pair of homologous chromosomes in the genome B, so two β subunits are synthesized per two α subunits. The nulli-tetrasomic NT3B-3A (4*α* + 2*β*)^2^ line lacks the third pair of chromosomes in diploid genome B. That pair was replaced with homeologous pair of chromosomes from the diploid genome A, so two β subunits are synthesized per four α subunits. Zymographic analysis of the AAT activities in these lines showed that bands staining intensity of the cytosolic AAT allozymes was different depending on the amount and proportion of α and β subunits synthesized by a particular wheat aneuploid line (Fig. 3). Due to the fact that Dt3AS (0*α* + 4*β*)^2^ line synthesizes only β subunits, there is only one visible band within AAT-3 zone at the AAT-3a position on the zymogram. The other used wheat aneuploid lines produce both α and β subunits, but in different proportions, and therefore the distribution of the bands staining intensity within AAT-3 zone is different for each line. Quantitative analysis of the scanned zymograms allowed to calculate the relative (expressed in  %) distribution of the AAT-3 allozymes activities in the studied aneuploid lines. Obtained experimental distribution values, together with theoretical distributions expected after substitution of various α and β values in the mathematical model for each line, are presented in Table 1. The greatest divergence (expressed as χ^2^ value) between experimental and theoretical distributions is obtained by substituting frequencies of α = 0.50 and β = 0.50 [17] in the mathematical model. It is the most noticeable for the Dt3BS and NT3B-3A line. In turn, when the frequencies of α = 0.62 and β = 0.38 [7] are substituted in the mathematical model, the divergences are much smaller. However, the highest degree of conformity between the experimental results and calculated theoretical results for all tested lines can be achieved by substituting α = 0.57 and β = 0.43 in the mathematical model. Thus, taking into consideration identical catalytic efficiency of all three AAT-3 allozymes [22], the results obtained in the present study show that the α subunit encoded by the genome A is synthesized more frequently than the β subunit encoded independently by the genomes B and D. This data may serve as a preliminary indication for the selection of strategies for obtaining synthetic wheat lines with increased AAT activity. Nevertheless, further studies regarding the contribution of the individual wheat genomes in AAT-3 subunits biosynthesis are necessary, and these should include real-time PCR method. In particular that the coding sequences of the TRAES3BF023000050CFD_g, traes_3AL_B137CC856 and traes_3DL_870617108 genes are currently known, and the small differences present in these sequences allow to analyze independently the α and β subunits transcript levels.

**Table 1:** A comparison of the theoretical and experimental distributions of the band staining intensities of cytosolic AAT allozymes activities in different aneuploid lines of common wheat (*Triticum* *aestivum*)

	Dt3AS (0*α* + 4*β*)^2^ (%)	M3A (1*α* + 4*β*)^2^ (%)	M3B (2*α* + 3*β*)^2^ (%)	Dt3AL (2*α* + 4*β*)^2^ (%)	Dt3BS (2*α* + 2*β*)^2^ (%)	NT3B-3A (4*α* + 2*β*)^2^ (%)
Experimental distributions—See Fig. 3						
AAT-3c (αα)	–	9.2	23.8	21.3	35.5	48.9
AAT-3b (αβ)	–	37.7	42.0	43.6	41.7	38.9
AAT-3a (ββ)	100.0	53.1	34.2	35.1	22.8	12.2
Theoretical distributions on the assumption that α = 0.50 and β = 0.50						
AAT-3c (αα)	–	4.0	16.0	11.0	25.0	44.5
AAT-3b (αβ)	–	32.0	48.0	44.5	50.0	44.5
AAT-3a (ββ)	100.0	64.0	36.0	44.5	25.0	11.0
χ^2^ = 33.2						
Theoretical distributions on the assumption that α = 0.56 and β = 0.44						
AAT-3c (αα)	–	5.8	21.1	15.1	31.3	51.5
AAT-3b (αβ)	–	36.6	49.7	47.5	49.3	40.5
AAT-3a (ββ)	100.0	57.6	29.2	37.4	19.4	8.0
χ^2^ = 12.5						
Theoretical distributions on the assumption that α = 0.57 and β = 0.43						
AAT-3c (αα)	–	6.2	22.0	15.9	32.5	52.7
AAT-3b (αβ)	–	37.4	49.8	47.9	49.0	39.8
AAT-3a (ββ)	100.0	56.4	28.2	36.2	18.5	7.5
χ^2^ = 12.2						
Theoretical distributions on the assumption that α = 0.58 and β = 0.42						
AAT-3c (αα)	–	6.6	23.0	16.7	33.6	53.9
AAT-3b (αβ)	–	38.2	49.9	48.3	48.8	39.0
AAT-3a (ββ)	100.0	55.2	27.1	35.0	17.6	7.1
χ^2^ = 12.8						
Theoretical distributions on the assumption that α = 0.62 and β = 0.38						
AAT-3c (αα)	–	8.4	27.2	20.2	38.4	58.6
AAT-3b (αβ)	–	41.2	49.9	49.5	47.1	35.9
AAT-3a (ββ)	100.0	50.4	22.9	30.3	14.5	5.5
χ^2^ = 24.9						

## Conclusions

The data presented in the present report specify that the genes encoding cytosolic AAT allozymes in common wheat (*Triticum aestivum* cv. Chinese Spring, 2n = 6x = 42, AABBDD) are localized on chromosomes: 3AL (0.42÷0.61), 3BL (0.38÷0.41) and 3DL (0.23÷0.81). Results of the quantitative analysis of the α and β subunit frequencies in six aneuploid lines of common wheat demonstrate that the individual participation of the diploid genome A is greater than the individual participation of the diploid genomes B and D in the biosynthesis of the subunits composing AAT-3 allozymes.
